# Structural Features and Digestibility of Corn Starch With Different Amylose Content

**DOI:** 10.3389/fnut.2021.692673

**Published:** 2021-06-21

**Authors:** Xinxin Lv, Yan Hong, Qiwei Zhou, Chengchen Jiang

**Affiliations:** ^1^Key Laboratory of Synthetic and Biological Colloids, Ministry of Education, Wuxi, China; ^2^School of Food Science and Technology, Jiangnan University, Wuxi, China; ^3^Qingdao Special Food Research Institute, ChangCheng Avenue, Qingdao, China; ^4^Collaborative Innovation Center for Food Safety and Quality Control, Jiangnan University, Wuxi, China

**Keywords:** *in vitro* digestibility, amylose, chain length distribution, crystal structure, corn starch

## Abstract

In this study, the *in vitro* digestibility of corn starch with different amylose content was determined. The results showed that waxy corn starch (WCS) and corn starch (CS) have the highest digestibility, while high amylose corn starch (HACS) has a higher content of resistant starch (RS). Besides being related to amylose content, RS content is also closely related to particle shape, debranched fine structure, molecular structure, and semi-crystalline structure. HACS can maintain a complete particle structure after gelatinization and enzymolysis; differential scanning calorimetry showed a positive correlation between gelatinization enthalpy and RS content. As the amylose content increased, the content of *fa* (DP 6–12) decreased, while the content of *fb2* (DP 25–36) and *fb3* (DP ≥ 37*)* increased, which in-turn decreased the cluster polymer formed by short branch chains, and the formation of more hydrogen bonds between long chain branches improved starch stability. D, which characterizes the compactness of starch semi-crystalline structure, increased with the increase of RS content. HACS 60 with the highest RS content had a unique surface fractal structure between 7.41 < *d* (2π*/q*) < 10.58 nm, indicating that the dense structure is effective in maintaining the RS content.

## Introduction

Starch is one of the most important carbohydrates in human life, with different applications in industries such as the food, petroleum, pharmaceutical, paint, and cosmetic industries, among others (1). Corn is the main botanical origin of starch production (2). Starch comprises three parts: rapidly digestible starch (RDS), slowly digestible starch (SDS), and resistant starch (RS) (3). RDS leads to a rapid increase in blood glucose levels after meals, which is harmful to diabetics; SDS releases glucose slowly, which can maintain stable blood glucose levels after meals. RS cannot be digested by enzymes in the small intestine, but can be fermented by microorganisms to produce short-chain fatty acids to promote beneficial intestinal flora and human health (4). Therefore, reducing the digestibility of starch is an effective strategy to prevent and control chronic diseases such as diabetes and obesity (5).

RS has been classified into five general subtypes: RS1–RS5 (6). Different types of RS have different mechanisms, RS2 refers to natural resistant starch granules such as high amylose corn starch (HACS), raw potato, and banana starch. HACS is of interest because of its health benefits and industrial uses (7). Understanding the resistance mechanism of HACS is an important step to regulate RS content. To the best of our knowledge, the structural features of starch that resist the action of digestive enzymes mainly include starch granules compactness, amylopectin/amylose ratio, amylopectin fine structures, molecular structure, crystalline structure, and semi-crystalline lamellae (8–13). Amylose can span the crystalline and amorphous regions of starch and connect the inside and outside of the granule. The high amylose content in HACS causes starch to form tightly packed granules inaccessible to digestive enzymes (14). The higher the amylose content, the more conducive is the formation of tightly arranged linear sequences, thereby reducing the damage to starch structure by heat treatment and reducing the rate of starch enzymatic hydrolysis (15, 16). To improve the starch resistance of HACS, researchers increased the amylose content through genetic breeding, and to change the amylose arrangement (17). The increase in amylose content will also bring about other structural changes. The effect of the fine structure of amylose on the function of HACS has been studied, and the molecular structure of starch is also very important to its overall physical and chemical properties. In the debranched chain length distributions, a higher proportion of long chains (DP ≥ 37) will increase the starch gelatinization temperature and reduce the peak viscosity (18, 19). However, short chains (DP 6–12) may cause imperfections by formation of crystallites. Starch with fewer short chains has better enzymatic resistance and heat stability. Small angle X-ray scattering (SAXS) analysis has been widely used to study the semi-crystalline structure of starches from different sources (12, 20–22) and different modified starches (23–25). The rapid development of SAXS analysis technology provides more possibilities for studying the semi-crystalline structure of HACS. The closer the semi-crystalline structure, the higher the thermal stability and resistance to the enzymatic degradation of starch.

HACS has always been an important RS, but the principle of amylose increasing starch RS content needs further study. This experiment studied the changes in particle shape, molecular structure, crystal structure and fractal structure brought about by amylose, and its influence on RS content. The results obtained from these studies were used to establish the correlation between structural features and *in vitro* digestibility, provide accurate target structures for gene breeding, and reduce the difficulty of gene breeding to regulate the RS content of corn starch.

## Materials and Methods

### Materials

Waxy corn starch (WCS, moisture content: 12.98%, amylose content: 3.23%) was purchased from COFCO Corporation (Jilin, China); Corn starch (CS, moisture content: 11.95%, amylose content: 20.13%), HACS 70 (moisture content: 13.09%, amylose content: 71.16%), and HACS 80 (moisture content: 12.38%, amylose content: 80.71%) were purchased from AnHui King Corn Agriculture Science and Technology Development Co., Ltd. (Anhui, China); HACS 50 (moisture content: 12.78%, amylose content: 51.22%) was purchased from Cargill Investment Co., Ltd. (Shanghai, China); HACS 60 (moisture content: 9.42%, amylose content: 61.26%) was purchased from Yiruian Food Ingredients Co., Ltd. (Shanghai, China); Pancreatin (porcine pancreas, 8 × USP), amyloglucosidase (*Aspergillus niger*, ≥ 300 U/mL), pepsin (porcine stomach mucosa, ≥ 250 U/mg), invertase (solids, ≥ 300 U/mg), and isoamylase (protein, 3 × 10^6^ U/mg) were purchased from Sigma-Aldrich (St. Louis, MI, USA); The glucose oxidase-peroxidase (GOPOD) kit was purchased from Beijing Leadman Biochemistry Co., Ltd. (Beijing, China); Other reagents were purchased from China National Medicines Co., Ltd. (Shanghai, China) and were of analytical grade.

### *In vitro* Digestibility

The method for determining the digestibility of starch is based on previous studies (3, 26). The released glucose content was measured using a GOPOD kit and calculated using Eq. (1):

(1) $$\begin{array}{r} glucose\%=\frac{\left(A_{t}-A_{b}\right)\times c\times V\times D}{A_{S}\times w}\times 100 \end{array}$$

Where *A*_*t*_ is the absorbance of the test solution at 520 nm; *A*_*b*_ is the absorbance of the blank solution at 520 nm; *c* is the concentration of the standard solution (mg glucose/mL, provided by the glucose oxidase-peroxidase kit); *V* is the total volume of the test solution; *D* is the dilution factor; *A*_*s*_ is the absorbance of the standard solution at 520 nm; *w* is the weight of the sample used for analysis (mg), which can be corrected for moisture content using Eqs. (2)–(4).

(2) $$\begin{array}{r} RDS=\left(G20-FG\right)\times 0.9 \end{array}$$

(3) $$\begin{array}{r} SDS=\left(G120-G20\right)\times 0.9 \end{array}$$

(4) $$\begin{array}{r} RS=TS-RDS-SDS \end{array}$$

Where FG is the free glucose content and TS is the total starch content (%, dry basis) of the sample.

### Polarizing Microscopy Analysis

Samples (0.2 mg, dry basis) were weighed, 2 mL of water was added, and the samples were gelatinized at 100°C for 30 min. The gelatinized samples were freeze-dried and dispersed in an aqueous solution (glycerin:water = 1:1). The polarized light birefringence cross was observed under a polarized light microscope (Olympus, Tokyo, Japan).

### Scanning Electron Microscopy

After the starch is gelatinized at 100°C, simulated digestion *in vitro* was performed. The products digested for 120 min were collected, ethanol precipitated and freeze-dried. According to the reported method (27), the corn starch with different amylose content and the collected digestion products were fixed and sprayed with gold, and the samples were observed and photographed under an acceleration voltage of 3 kV.

### Rapid Viscosity Analysis

The pasting properties of the HACS were determined by rapid viscosity analysis (RVA) using a Tech Master analyzer (23) (Perten, Stockholm, Sweden). Each complex was suspended in deionized water (10%, w/w, dry basis) in an RVA aluminum box, the samples were heated from 50 to 140°C in 9 min, then kept at 140°C for 2 min, and finally dropped from 140 to 50°C in 9 min.

### Differential Scanning Calorimetry

The thermal properties were determined by differential scanning calorimetry (DSC) using the method already reported (28). The temperature range was 50–160°C, and the heating rate was 10°C/min.

### Debranched Chain Length Distributions

The debranched chain length distributions of samples were determined using the method already reported (29), with some modifications. Samples (10 mg, dry basis) were dissolved in 2 mL citrate-disodium hydrogen phosphate buffer solution (pH = 3.5) and gelatinized at 100°C for 30 min. After the solution was cooled to 40°C, 100 μL isoamylase solution was added, and the reaction was conducted for 24 h at 40°C. The reaction was stopped by incubating for 20 min in a boiling water bath. Samples were then allowed to cool to room temperature and centrifuged at 13,000 g for 10 min. The supernatants were filtered through a 0.45 mm nylon microporous membrane to measure the chain length distributions using High Performance Anion Exchange Chromatography-Pulsed Amperometric Detector (Thermo, Waltham, Massachusetts, USA).

### Gel Permeation Chromatography

The weight-average molecular weight of the samples were measured using gel permeation chromatography (GPC) according to a previous method (30). After peak fitting, GPC data were used to characterize the weight-average molecular weight (M_w_) of the samples.

### X-Ray Diffraction

According to the method already reported (31), the crystal structure was determined using an X-ray diffractometer (Bruker AXS Ltd., Leipzig, Germany), and the relative crystallinity was calculated using the MDI Jade 5.0 software.

### Small Angle X-Ray Scattering Analysis

The semi-crystalline structure was determined by Small angle X-ray scattering (SAXS) analysis (32). SAXS measurements were conducted on SAXSess small angle X-ray scattering system (Anton Paar, Graz, Austria) which was equipped with a PW3830 X-ray generator (PANalytical) using Cu Kα radiation (λ = 0.1542 nm).

### Statistical Analysis Methods

The data were statistically analyzed using IBM SPSS Statistics v20 software (IBM, Armonk, NY, USA). Results were expressed as mean ± standard deviation.

## Results and Discussion

### *In vitro* Digestibility

**Figure 1:**
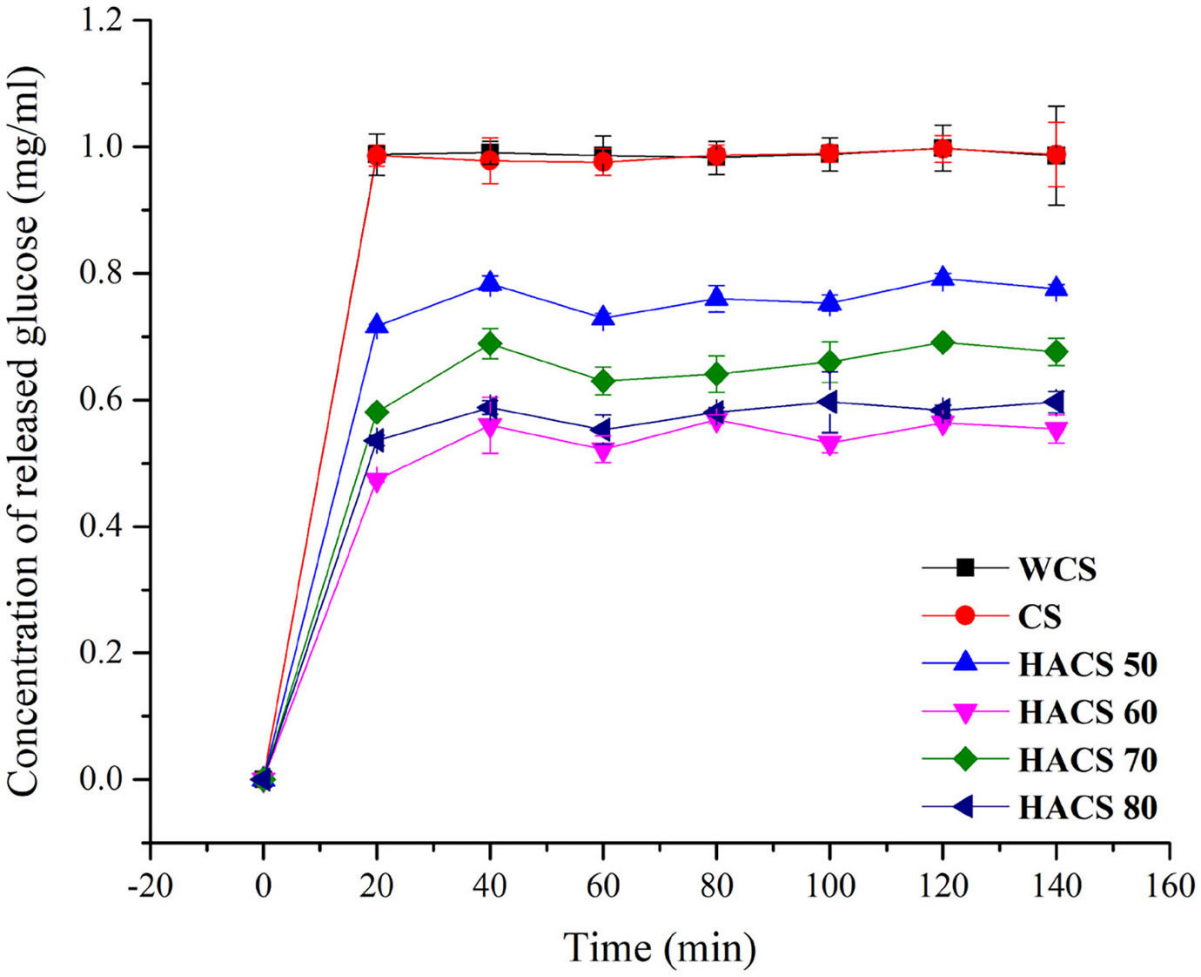
*In vitro* digestibility of corn starch with different amylose content.

The results of the *in vitro* digestive properties of corn starch with different amylose content are shown in Figure 1. During the 150 min digestion process, according to the slope of the glucose release curves, samples were all digested rapidly within 20 min of enzymatic hydrolysis, whereas the digestion rate within 20–150 min increased slowly. The *in vitro* digestibility of HACS ranged from 45 to 80%, indicating that HACS has strong digestion resistance. The RS content values of RDS, SDS, and RS for the samples are listed in Table 1. The RDS content of WCS and CS was ~88%. In comparison, the HACS with over 50% amylose content showed higher RS content. With the increase in amylose content, the RS content of HACS 50, HACS 70, and HACS 80 also gradually increased. There is a positive correlation between amylose content and RS content (33), which is mainly because amylose is not easily digested by enzymes (34).

**Table 1:** RDS, SDS, RS content of corn starch with different amylose content.

**Samples**	**RDS (%)**	**SDS (%)**	**RS (%)**
WCS	88.92 ± 0.83^e^	1.80 ± 0.08^a^	9.28 ± 0.75^b^
CS	88.74 ± 0.47^e^	3.49 ± 0.13^b^	7.01 ± 0.34^a^
HACS 50	65.00 ± 0.23^d^	6.64 ± 0.39^d^	28.35 ± 0.17^c^
HACS 60	43.76 ± 0.76^a^	7.13 ± 0.45^d^	49.10 ± 0.31^f^
HACS 70	52.13 ± 0.24^c^	9.47 ± 0.40^e^	38.40 ± 0.18^d^
HACS 80	48.38 ± 0.31^b^	4.28 ± 0.24^c^	47.34 ± 0.14^e^

*Values followed by different superscripts in the same column are significantly different (p < 0.05)*.

It is worth noting that the sample with the highest RS content is HACS 60, which has an RS content of 49.10%. This result shows that besides the amylose content, some factors affect the RS content of starch (9). When determining starch digestibility, researchers found HACS retains some intact granules after gelatinization. Therefore, to further explore the factors affecting the RS content, gelatinized HCAS particles were observed.

### Particle Integrity

#### Cross-Polarization

**Figure 2:**
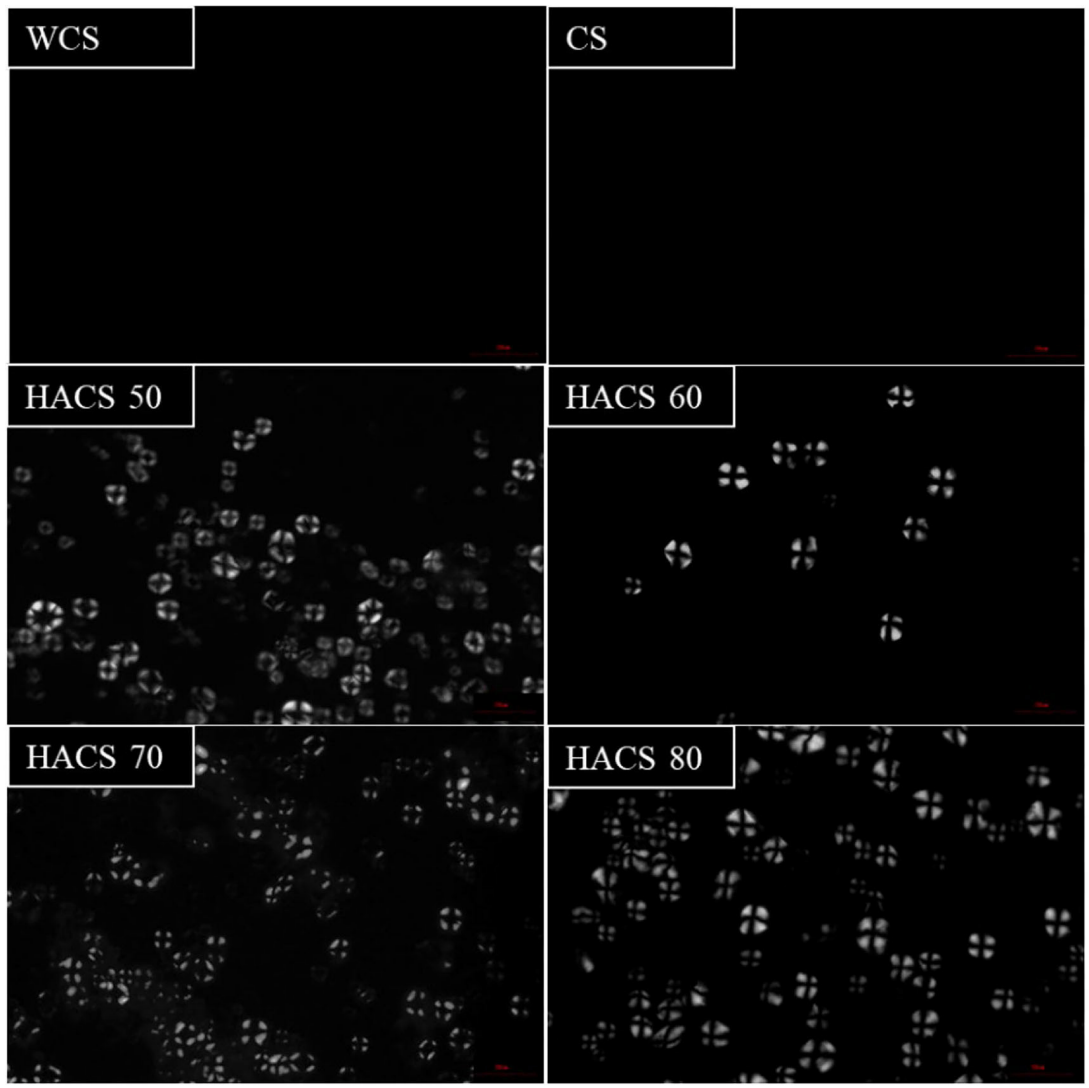
The cross-polarization of corn starch with different amylose content after gelatinization.

The orderly arranged starch molecules of crystalline regions and disorderly arranged starch molecules in amorphous regions normally generate an anisotropic phenomenon in starch granules, leading to a birefringence cross under polarized light (35). The gelatinized corn starch granules with different amylose content were analyzed (Figure 2). Figure 2 shows that after gelatinization, HACS still maintains cross-polarization, indicating that the internal structure of the starch granules with an amylose content >50% [meet the definition of high amylose starch (7)] was tight, and the arrangement of amylose and amylopectin was not completely destroyed. The internal structure of HACS was compact and could refrain the particles from breaking under high temperature, and would reduce the water holding capacity of starch, thus reducing the viscosity of starch after gelatinization and starch RDS content. Therefore, the gelatinization properties of HACS were measured.

#### Rapid Viscosity Analysis of Pasting Properties

**Figure 3:**
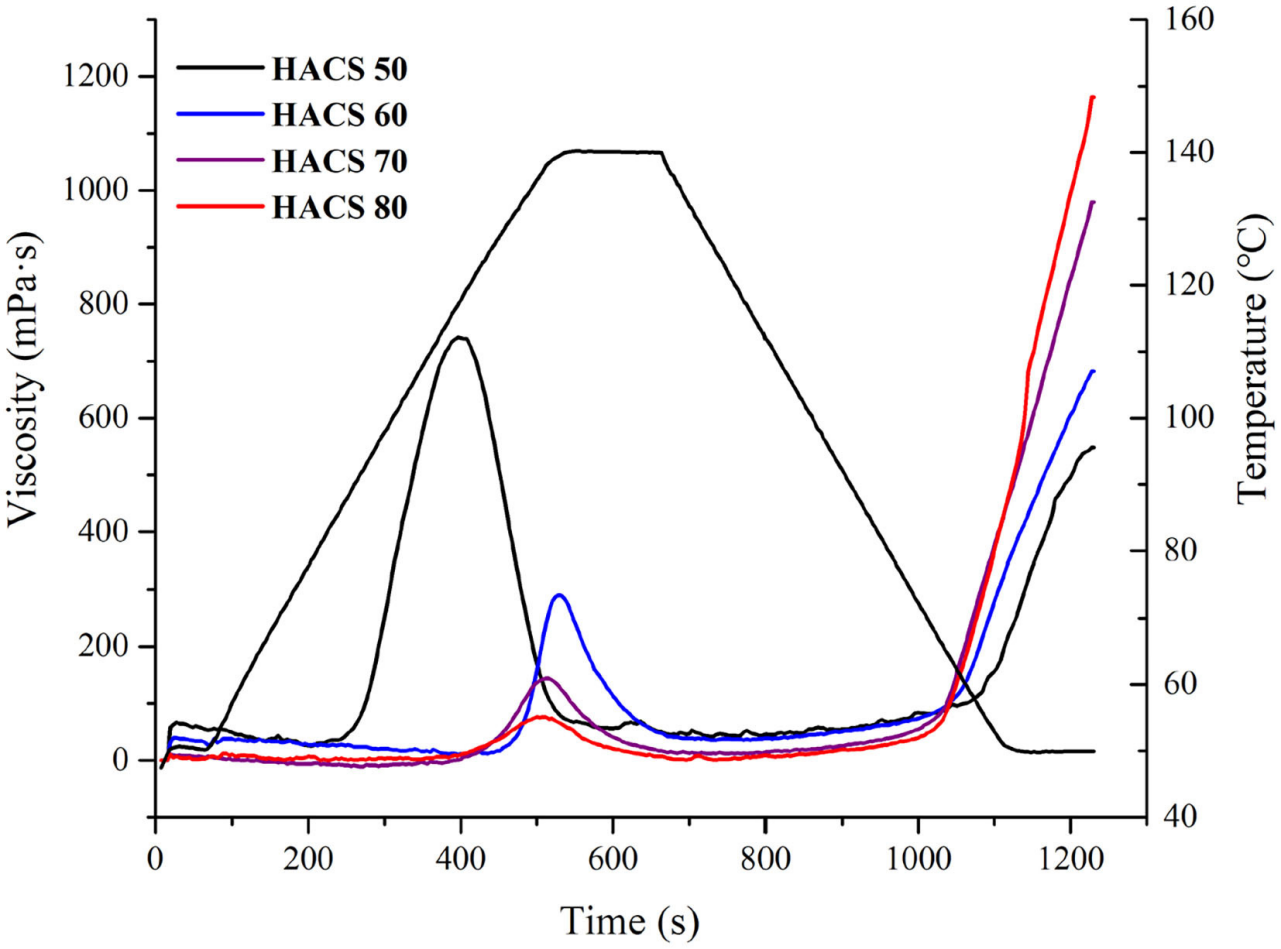
RVA pasting profiles of HACS with different amylose content.

Under excessive water environment and heating conditions, starch granules swell and rupture after being absorbed by water, causing the viscosity of the sample to increase. Observing the starch gelatinization process helps to understand the breakdown of starch granules (36). As seen in Figure 3, the peak viscosity of HACS decreased with an increase in the amylose content, which indicates that increased amylose content makes it more difficult for the granules to break (37). This result is consistent with the cross-polarization after HACS gelatinization. Setback viscosity indicates starch retrogradation during the cooling period, and the final viscosity reflects the viscosity of cold starch paste, both of which increased with the increase in amylose content (Table 2); HACS 80 had the highest final (1,164 mPa·s) and setback (1,163 mPa·s) viscosities.

**Table 2:** Gelatinization characteristic value of corn starch with different amylose content.

**Samples**	**Peak viscosity (mPa·s)**	**Breakdown (mPa·s)**	**Final viscosity (mPa·s)**	**Setback (mPa·s)**
WCS	3507.7 ± 1.5^f^	2455.7 ± 1.5^f^	1487.0 ± 1.0^e^	154 ± 1.0^a^
CS	3356.7 ± 2.1^e^	1258.3 ± 1.5^e^	3927.0 ± 1.7^f^	434 ± 0.6^b^
HACS 50	741.6 ± 1.5^d^	699.7 ± 1.5^d^	548.0 ± 2.0^a^	506 ± 1.2^c^
HACS 60	290.0 ± 1.0^c^	278.3 ± 0.6^c^	681.0 ± 1.0^b^	670 ± 0.5^d^
HACS 70	144.0 ± 1.0^b^	132.0 ± 1.0^b^	978.7 ± 1.5^c^	967 ± 1.0^e^
HACS 80	76.4 ± 0.5^a^	76.2 ± 1.3^a^	1164.3 ± 1.5^d^	1163 ± 2.1^f^

*Values followed by different superscripts in the same column are significantly different (p < 0.05)*.

#### Starch Granules

**Figure 4:**
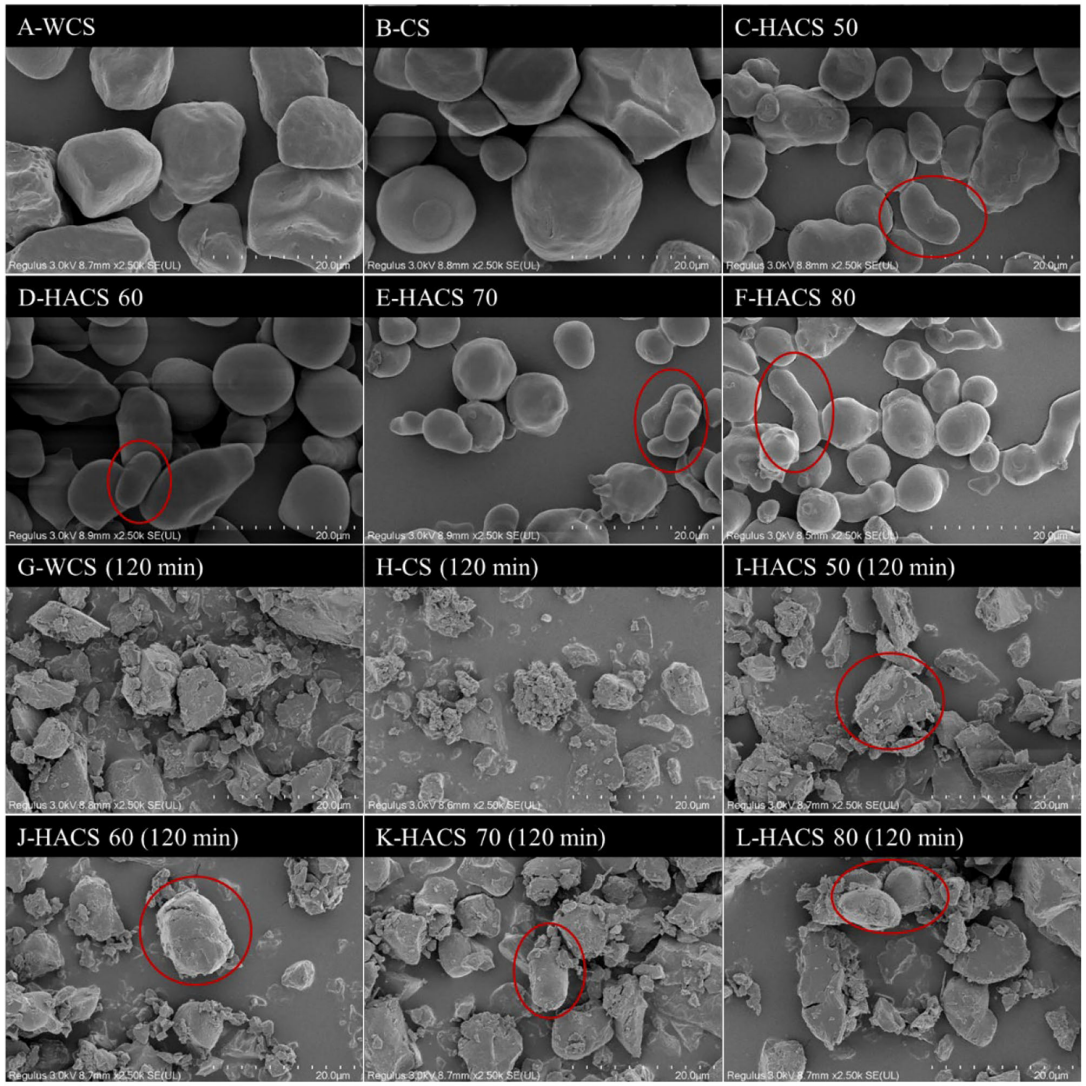
The morphology of corn starch with different amylose content **(A–F)** and the morphology of corn starch **(G–L)** after enzymolysis for 120 min.

It can be seen from Figures 4A–F that the WCS and CS particles are polygonal, while HACS has some spherical granules and rod/filamentous granules, which is consistent with previous reports (38). The granular structure of HACS granules contributed to the resistance of starch molecules to pancreatic α-amylase hydrolysis. Amylose increases the tightness of starch granules, so starch granules still exist after HACS enzymatic hydrolysis for 120 min (Figures 4G–L), while WCS and CS can only observe broken gel pieces. This is mainly due to the presence of long-chain double helix microcrystals derived from amylose and intermediate components (IC) that stabilize the starch structure (38).

### Thermal Properties

Values corresponding to various thermodynamic properties of corn starch with different amylose content are presented in Table 3. DSC results demonstrated that the onset (initial) gelatinization temperature (*T*_*o*_), peak gelatinization temperature (*T*_*p*_), and conclusion (termination) temperature (*T*_*c*_) of HACS were significantly delayed, and the gelatinization temperature range (*T*_*c*_–*T*_*o*_) increased significantly. These results concur with those reported for WCS (39). The inner area of starch granules mainly comprises loosely packed amylopectin growth rings with semi-crystalline flakes, which are fragile under gelatinization or hydrolysis (40), therefore, as the content of amylopectin decreased, the gelatinization enthalpy (Δ*H*) of HACS increased, the maximum Δ*H* was 18.24 J/g. Comparing the content of resistant starch in Table 1, the study found that with the increase in RS content, the *T*_*o*_, *T*_*p*_, *T*_*c*_, and Δ*H* of HACS showed a consistent increasing trend, which indicates that the RS content of starch is closely related to the thermal stability of starch. Besides amylose content, starch digestibility is still affected by the orderly spiral structure between amylose molecules (41). The orderly arrangement of amylose could increase the stability of starch granules and improve heat resistance. HACS 60 had the highest RS content (49.10%) and the highest Δ*H*, proving that it has a more ordered spiral structure between amylose molecules.

**Table 3:** Thermodynamic parameters of corn starch with different amylose content.

**Samples**	***T_***o***_* (^**°**^C)**	***T_***p***_* (^**°**^C)**	***T_***c***_* (^**°**^C)**	***T_***c***_*–*T_***o***_* (^**°**^C)**	***ΔH* (J/g)**
WCS	67.37 ± 0.05^c^	71.56 ± 0.09^a^	78.62 ± 0.05^a^	11.25	14.54 ± 0.36^b^
CS	67.08 ± 0.21^b^	71.86 ± 0.17^b^	79.05 ± 0.27^b^	11.97	13.14 ± 0.12^a^
HACS 50	62.55 ± 0.14^a^	75.33 ± 0.03^c^	103.05 ± 0.16^c^	40.50	16.86 ± 0.05^c^
HACS 60	92.94 ± 0.23^f^	95.44 ± 0.14^e^	120.73 ± 0.13^f^	27.79	18.24 ± 0.15^e^
HACS 70	76.58 ± 0.12^d^	94.06 ± 0.05^d^	104.02 ± 0.12^e^	27.44	17.75 ± 0.13^d^
HACS 80	84.49 ± 0.15^e^	94.94 ± 0.24^d^	106.26 ± 0.17^d^	21.77	18.05 ± 0.12^e^

*Values followed by different superscripts in the same column are significantly different (p < 0.05)*.

### Debranched Chain Length Distributions

According to existing report (42), the debranched chain segment can be divided into four parts. As shown in Table 4, as the apparent amylose content increases, the average chain length of starch and the degree of branching increases (43), the maximum average chain length is 22.85 and the minimum branching degree is 0.215. The high content of long chains (*fb3*, DP ≥ 37) and low degree of branching [*fa*/(*fb1* + *fb2* + *fb3*)] in amylopectin are associated with the digestibility of starch. The long chains in amylopectin can form longer double helices and strengthen the hydrogen bonding force between the chain segments, making the structure more stabilized and increasing the RS content of starch. However, the short branches of amylopectin make the layered structure of the crystal unstable.

**Table 4:** Debranched chain length distributions and weight-average molecular weight of corn starch with different amylose content.

**Samples**	**Average chain length**	**Branch degree**	**Amylopectin branch chain length distribution (%)**				**M**_****w****_	
		***fa/*(*fb1* + *fb2* + *fb3*)**	***fa* (%)**	***fb1* (%)**	***fb2* (%)**	***fb3* (%)**	**Peak 1**	**Peak 2**
WCS	19.15 ± 0.01^a^	0.358	26.25 ± 0.09^f^	51.00 ± 0.21^d^	14.97 ± 0.06^a^	7.23 ± 0.08^a^	2.23 × 10^7^	-
CS	19.47 ± 0.02^b^	0.339	25.14 ± 0.09^e^	50.58 ± 0.05^c^	15.93 ± 0.05^b^	7.63 ± 0.07^b^	1.37 × 10^7^	2.25 × 10^5^
HACS 50	20.50 ± 0.05^c^	0.258	20.38 ± 0.06^d^	53.05 ± 0.01^e^	16.28 ± 0.27^c^	9.50 ± 0.03^c^	1.11 × 10^7^	4.28 × 10^5^
HACS 60	20.74 ± 0.04^d^	0.257	20.10 ± 0.08^c^	50.45 ± 0.47^c^	17.82 ± 0.03^d^	10.14 ± 0.04^d^	5.94 × 10^6^	2.59 × 10^5^
HACS 70	22.51 ± 0.09^e^	0.215	17.46 ± 0.04^a^	47.82 ± 0.17^b^	19.24 ± 0.05^e^	14.17 ± 0.07^e^	–	6.42 × 10^5^
HACS 80	22.85 ± 0.04^f^	0.225	17.90 ± 0.04^b^	45.44 ± 0.05^a^	19.10 ± 0.02^e^	15.02 ± 0.10^f^	–	5.03 × 10^5^

*Values followed by different superscripts in the same column are significantly different (p < 0.05)*.

### Weight-Average Molecular Weight

**Figure 5:**
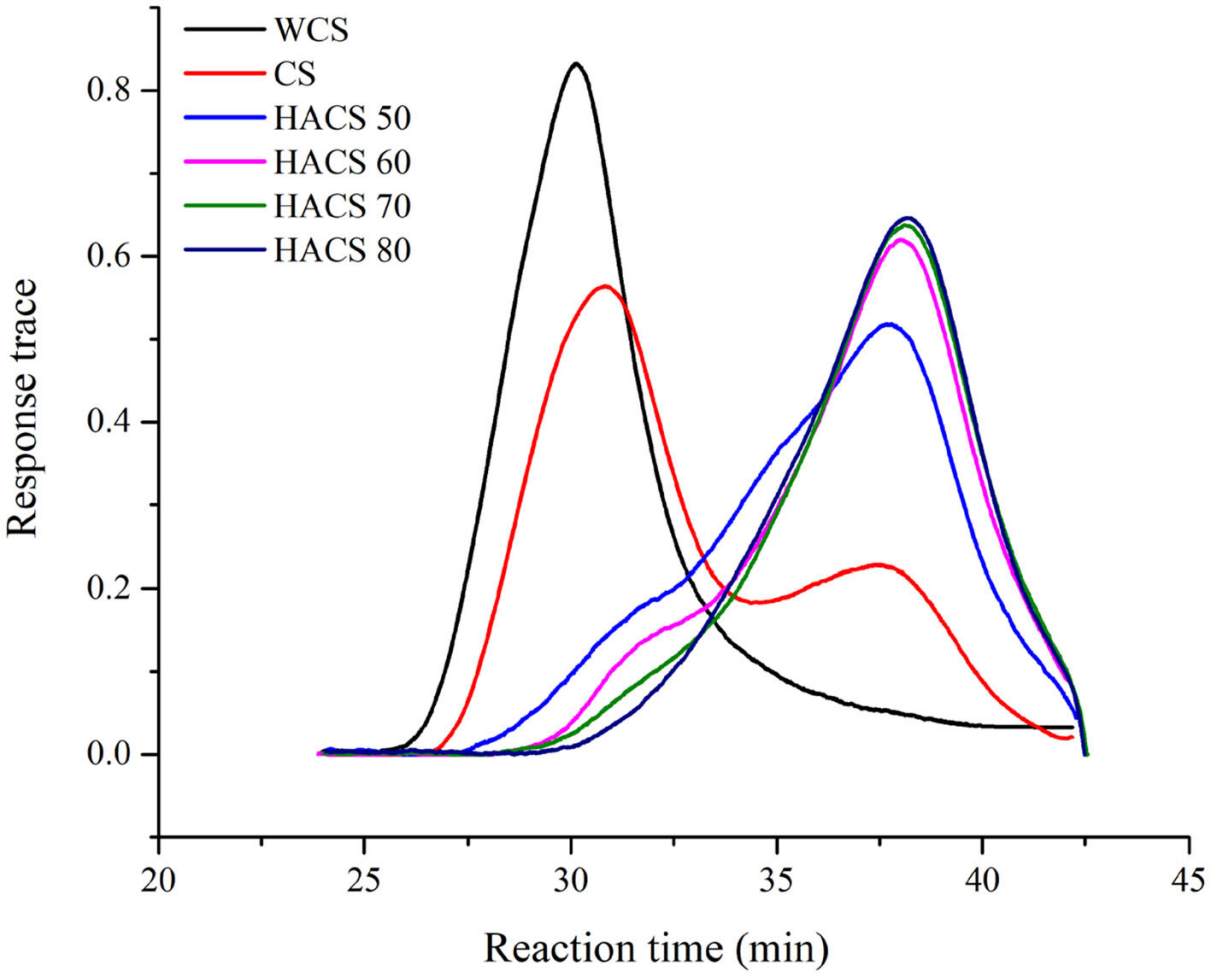
Weight-average molecular weight of corn starch with different amylose content.

Table 4 shows that as the amylose content increased, the Weight-average molecular weight (M_w_) of the samples continued to decrease. The maximum and minimum M_w_ of the samples were 2.23 × 10^7^ and 5.03 × 10^5^, respectively. HACS obviously had a narrow M_w_ (Figure 5), which characterized the subtle differences between starch molecules and the relatively uniform molecular size (29). Amylose mainly comprises a linear structure (glucose monomers with α-1,4-glycosidic bonds) and several long branches. According to existing research, during the digestion process, the long straight chains of CS are broken into short straight chains, and the original RS content of the starch can be increased through the formation of more double helices (43). A large amount of amylose in HACS easily produces a similar structure during digestion, which may be an important reason why HACS has a high RS content.

### Crystal Structure

**Figure 6:**
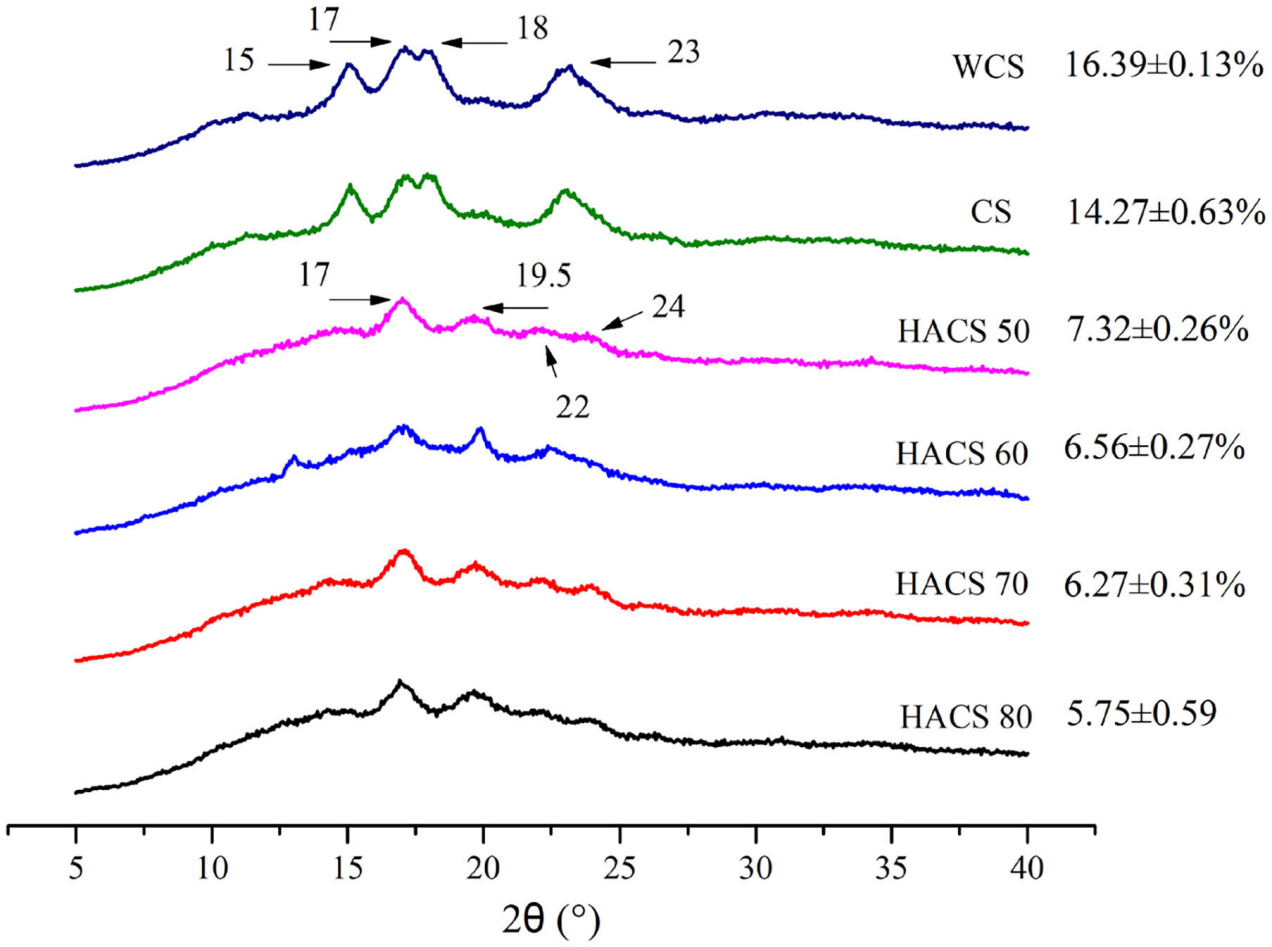
Crystal structure of corn starch with different amylose content.

Figure 6 shows the X-ray diffraction (XRD) pattern and relative crystallinity of corn starch with different amylose content. The crystal structure of starch can be attributed to the accumulation of amylopectin side chains forming a double helix (44), which can be divided into A, B, and C types; HACS belongs to type B starch. The relative crystallinity of HACS is lower than WCS and CS, and as the content of amylose increases, the relative crystallinity gradually decreases. Compared with type A starch, the content of *fa* (DP6-12) was lower, and the content of *fb3* (DP ≥ 37) was higher, which is consistent with the results in Table 4. It can be seen that the crystal structure does not promote the increase of RS content.

### Fractal Structure

**Figure 7:**
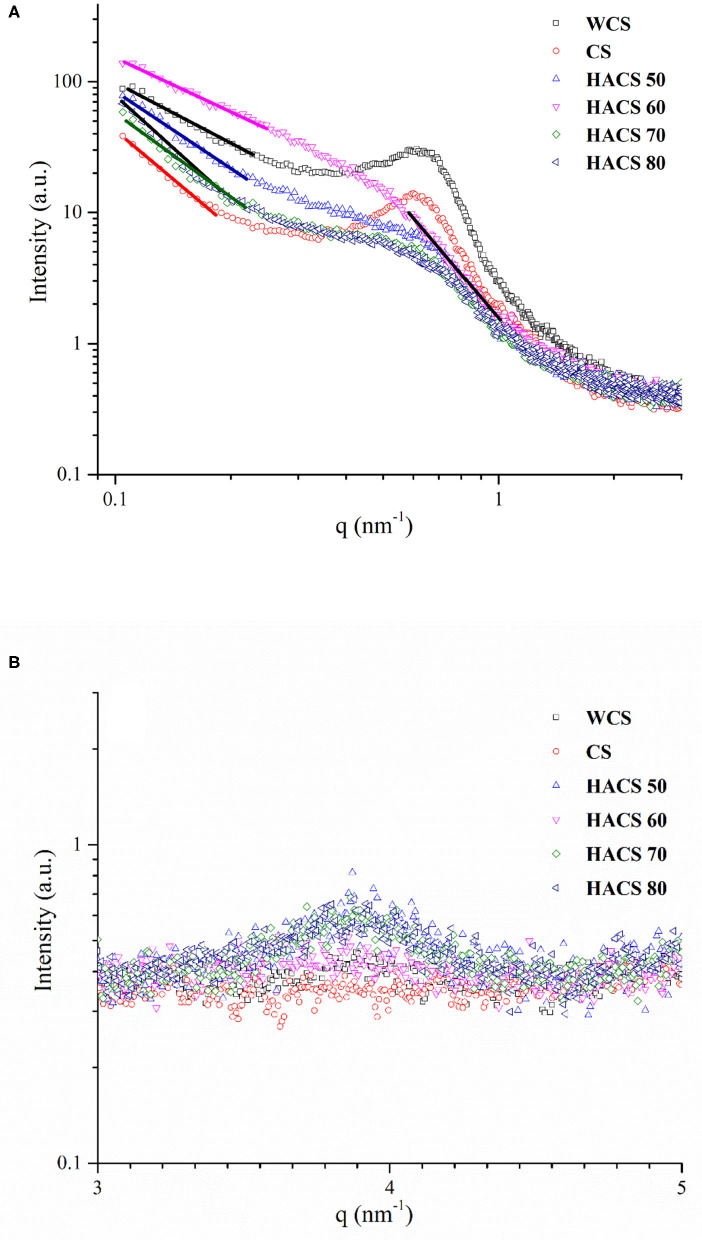
Double-logarithmic SAXS patterns **(A)**, log I-q SAXS patterns **(B)**.

The double logarithmic SAXS pattern of corn starch with different linear contents is shown in Figure 7A. According to the scattering power law equation: *I* = *q*^−α^, where *I* is the SAXS intensity and α is the index which can calculate the *D* value of the surface/mass fractal structure, and can be obtained from the slope of the log-log SAXS diagram. When 1 < α < 3, the scatterer is a mass fractal, which is relatively loose. For fractal index *D*_*m*_ = α, the closer *D*_*m*_ is to 1, the looser it is, and the closer to 3 the denser; when 3 < α < 4, the scatterer is a surface fractal. Relatively dense, rough surface, the fractal index *D*_*s*_ = 6–α is generally between 2 and 3. The closer *D*_*s*_ is to 2, the smoother the surface, whereas the closer to 3, the rougher the surface. According to the Woolf-Bragg formula *d* = 2π/*q*, the scattering peak of about 0.6 nm^−1^ in the SAXS curve can calculate the semi-crystalline layer thickness d of starch granules. The scattering object of the surface fractal is more compact than the scattering object of the mass fractal.

HACS 60 has a shoulder-like structure at *q* = 0.2–0.4 nm^−1^, which may be caused by the tight rearrangement of amylose (45), which may have been likely because it had two fractal-scattering regions at high and low *q* values, with different fractal dimensions. According to fractal geometry, scattering patterns with multiple power law regions can indicate the unique structural characteristics of materials at different length scales (46). As presented in Figure 7A and Table 5, all samples belong to the mass fractal. Samples WCS (*D*_*m*1_ = 1.07) and CS (*D*_*m*1_ = 1.01) were characterized between 17.20 < *d* (2π/*q*) < 43.77 nm. HACS 50 (*D*_*m*1_ = 1.28), HACS 70 (*D*_*m*1_ = 1.11), and HACS 80 (*D*_*m*1_ = 1.16) were characterized between 17.20 < *d* (2π/*q*) < 37.04 nm. HACS 60 (*D*_*m*1_ = 1.44) was characterized between 12.67 < *d* (2π/*q*) < 57.09 nm. These data indicate that the scattering objects in HACS 60 samples are more compact and the mass fractal structure is formed in a large range. Interestingly, for HACS 60, the surface fractal structure is observed to be between ~7.41 < *d* (2π/*q*) < 10.58 nm, and the fractal dimension *D*_*s*2_ = 2.46, which indicates that the scattering objects at the lower scale level are dense and smooth surfaces. As shown in Figure 7B HACS has a diffraction peak around *q* = 3.5, which is a unique phenomenon of type B crystals (47). This follows the phenomenon observed shown in XRD analysis.

**Table 5:** SAXS parameters of different samples.

**Samples**	***q* (nm^**−1**^)**	***d*_**1**_ (nm)**	*****α**_1_***	***D_***m*1**_***	*****α**_2_***	***D_***s*2**_***
WCS	0.6130	10.24	1.07	1.07	–	–
CS	0.6000	10.47	1.01	1.01	–	–
HACS 50	0.5908	11.20	1.28	1.28	–	–
HACS 60	–	–	1.44	1.44	3.54	2.46
HACS 70	0.5935	10.58	1.11	1.11	–	–
HACS 80	0.5869	10.70	1.16	1.16	–	–

## Conclusion

In this study, we determined the *in vitro* digestibility of corn starch with different amylose content. In addition, the factors affecting digestibility were discussed, including particle morphology, debranched chain fine structure, molecular structure, and semi-crystalline structure. The results showed that the higher the amylose content, the higher the RS content. The lower the *fa* content, the higher the *fb2* and *fb3* content, and the more stable the starch structure, the higher the RS content. In addition to amylose content, the arrangement of amylose also has an important influence on RS content. In the quality fractal, the larger the *D*_*m*_, the higher the RS content. The shoulder structure is the special structural factor contributing to the highest resistance of HACS 60, and the dense surface fractal is also one of the biggest factors for resistance in starch. Understanding the relationship between the structural factors affecting HACS resistance and RS content will help us to further cultivate corn starch with specific RS content.

## Data Availability Statement

The original contributions generated for the study are included in the article/supplementary material, further inquiries can be directed to the corresponding author/s.

## Author Contributions

XL: experimental arrangement and writing. YH: conceptualization, methodology, and supervision. QZ and CJ: analysis and literature review.

## Conflict of Interest

The authors declare that the research was conducted in the absence of any commercial or financial relationships that could be construed as a potential conflict of interest.
